# Treatment selection in multi-arm multi-stage designs: With application to a postpartum haemorrhage trial

**DOI:** 10.1177/17407745221136527

**Published:** 2023-01-17

**Authors:** Babak Choodari-Oskooei, Soe Soe Thwin, Alexandra Blenkinsop, Mariana Widmer, Fernando Althabe, Mahesh KB Parmar

**Affiliations:** 1 MRC Clinical Trials Unit at UCL, Institute of Clinical Trials and Methodology, University College London (UCL), London, UK; 2 Maternal and Perinatal Health Unit, Department of Sexual and Reproductive Health and Research (SRH), World Health Organization (WHO), Geneve, Switzerland; 3 Amsterdam Institute for Global Health and Development, Amsterdam, Netherlands

**Keywords:** Phase III selection designs, adaptive trial designs, familywise type I error rate, FWER, PWER, RED trial, MAMS

## Abstract

**Background:**

Multi-arm multi-stage trials are an efficient, adaptive approach for testing many treatments simultaneously within one protocol. In settings where numbers of patients available to be entered into trials and resources might be limited, such as primary postpartum haemorrhage, it may be necessary to select a pre-specified subset of arms at interim stages even if they are all showing some promise against the control arm. This will put a limit on the maximum number of patients required and reduce the associated costs. Motivated by the World Health Organization Refractory HaEmorrhage Devices trial in postpartum haemorrhage, we explored the properties of such a selection design in a randomised phase III setting and compared it with other alternatives. The objectives are: (1) to investigate how the timing of treatment selection affects the operating characteristics; (2) to explore the use of an information-rich (continuous) intermediate outcome to select the best-performing arm, out of four treatment arms, compared with using the primary (binary) outcome for selection at the interim stage; and (3) to identify factors that can affect the efficiency of the design.

**Methods:**

We conducted simulations based on the refractory haemorrhage devices multi-arm multi-stage selection trial to investigate the impact of the timing of treatment selection and applying an adaptive allocation ratio on the probability of correct selection, overall power and familywise type I error rate. Simulations were also conducted to explore how other design parameters will affect both the maximum sample size and trial timelines.

**Results:**

The results indicate that the overall power of the trial is bounded by the probability of ‘correct’ selection at the selection stage. The results showed that good operating characteristics are achieved if the treatment selection is conducted at around 17% of information time. Our results also showed that although randomising more patients to research arms before selection will increase the probability of selecting correctly, this will not increase the overall efficiency of the (selection) design compared with the fixed allocation ratio of 1:1 to all arms throughout.

**Conclusions:**

Multi-arm multi-stage selection designs are efficient and flexible with desirable operating characteristics. We give guidance on many aspects of these designs including selecting the intermediate outcome measure, the timing of treatment selection, and choosing the operating characteristics.

## Introduction

Efficient phase III clinical trial designs are needed to speed up the evaluation of new therapies. Multi-arm multi-stage (MAMS) randomised clinical trial designs have been developed to achieve this goal with two main components: the multi-arm aspect allows multiple experimental arms to be compared to a common control in a single trial; the multi-stage aspect allows interim analyses so that recruitment to the experimental arms which are performing no better than control can be stopped before the planned end of the study. This allows multiple research questions to be answered under the same protocol and in the confirmatory setting.

Royston et al.^1,2^ developed a framework for MAMS designs that allows the use of an intermediate ( I ) outcome at the interim stages that may or may not be the same as the definitive ( D ) outcome at the final analysis. This further increases the efficiency of the MAMS design by stopping recruitment to treatment arms for lack-of-benefit at interim stages based on an I outcome. Using an I outcome also allows interim analyses to be conducted sooner, so recruitment to poorly performing arms can be stopped earlier than if using the primary outcome throughout. The design has been extended to include interim stopping boundaries for overwhelming efficacy on the primary outcome.^3.^Choodari-Oskooei et al.^4^ give an extensive account of Royston et al.’s MAMS designs and discuss their underlying principles. In this article, the MAMS designs which utilise the *I* outcome at the interim looks are denoted by I≠D . Designs that monitor all the arms on the same definitive outcome throughout the trial are denoted by I=D . For simplicity, we consider the original design with only lack-of-benefit stopping boundaries with an I outcome in this article.

In a general MAMS design, all arms can reach their final stage of recruitment if they pass each interim analysis. As a result, the number of experimental arms recruiting at each stage cannot be pre-determined. Therefore, the actual sample size of the trial can be varied considerably with its maximum being when all the treatment arms reach the final stage. To distinguish this setting from the pre-specified treatment selection setting, we call it ‘full’ MAMS. In such trials with a large number of experimental arms, the maximum sample size might be too large to achieve or for any agency to fund it. In these cases, it may be more appropriate to pre-specify the number of experimental arms that will be taken to each stage, alongside a criterion for selecting them. One example of such design is the Refractory HaEmorrhage Devices (RED) trial in postpartum haemorrhage (PPH).

This is the first presentation of MAMS ‘selection’ designs within the general MAMS framework introduced by Royston et al. In the MAMS selection design, the selection of research arms after the interim analysis can be made based on the ranking of treatment effects or a combination of the rankings and safety results. Traditionally, the selection of the treatments has been made in phase II trials where strict control of operating characteristics is not a concern. In a MAMS selection design, the selection and confirmatory stages are implemented within one trial protocol, and the selection of the most promising treatments can be made at multiple stages. Patients will be randomised from the start to all the experimental and control arms, and the primary analysis of the experimental arms that reach the final stage includes all randomised individuals from all selection stages.

MAMS selection designs can reduce maximum sample size and simplify planning. It is, however, unclear how the design parameters should be chosen to maintain these benefits when strong control of operating characteristics is required. Given the above constraints and the MAMS design framework, we explore (1) what is the best timing for treatment arm selection, (2) how to select treatments, (3) what is the impact of treatment selection based on an intermediate outcome on the operating characteristics of the design and (4) how does a MAMS selection design sample size compare with an optimal full MAMS design. When comparing multiple eligible designs, different optimality criteria can be used to choose between these designs.^5^ In this study, we focus on optimising power, by choosing designs which result in the earliest treatment selection time and lowest maximum sample size. This can be thought of as a minimax criterion – minimising the maximum sample size.^5^

Next, we introduce the RED trial and set out design challenges in trials in PPH.

## An example: RED trial

### Trial setting

PPH is one of the leading causes of global maternal morbidity and mortality. Despite recent advances in maternal health, death from PPH remains highly prevalent, accounting for nearly 70,000 maternal deaths worldwide every year.^6^ The RED trial is a phase III randomised clinical trial that uses the MAMS selection design investigating the efficacy of three different tamponade devices (the Ellavi fixed-volume uterine balloon tamponade (UBT), the Ellavi free-flow UBT, and the Suction Tube Uterine Tamponade (STUT) device) against the control device, the Foley catheter. The trial starts with randomisation at equal allocation ratio to three research arms and a control. One research arm is selected at stage 1 to continue to stage 2 with the control.

### Trial outcome and outcome measure

The primary outcome in this trial at both interim and final analyses is the binary (composite) outcome of PPH-related maternal mortality or invasive surgical procedures (MMS) up to 3 days postpartum. The trial is powered to detect a risk reduction in each experimental arm by 5% (absolute) and 33% (relative), that is, an event rate of 10% in each experimental arm and 15% in the control arm.

### Design challenges

Given the relatively low event rate of atonic refractory PPH in the target population, hundreds of thousands of deliveries would need to be screened to recruit a sufficient sample for a phase III trial on refractory PPH.^7^ As an example, 426,000 vaginal births would need to be screened to recruit 1366 participants who are required for a conventional two-arm parallel-group randomised controlled trial (RCT) with (one-sided) 2.5% significance level and 80% power to detect 5% absolute risk reduction from the control arm event rate of 15%.

A MAMS design with the similar pairwise type I error rate and power is more efficient than concurrent two-arm trials. However, in some MAMS designs such as the RED trial the familywise type I error rate (FWER) should be controlled at the pre-specified level (2.5%, one-sided) which then increases the required sample size for a given power.^8^ Freidlin et al.^9^ and Proschan and Waclawiw^10^ provide further guidance on this important design consideration. In the RED trial, the FWER was the overall type I error rate of interest – which should be controlled at 2.5% level (one-sided), since the interventions in two of the research arms were similar. Despite multiplicity correction, the ‘optimal’ MAMS design reduces the maximum sample size considerably when compared with three separate two-arm trials, by around 23%. (Note that the overall significance level of three independent two-arm trials, each with a 2.5% significance level, is about 3 times, that is, 7.3% = $1-(1-0.025{)}^{3}$ , the optimal MAMS design.) By ‘optimal’ MAMS designs, we mean the most efficient feasible designs, called admissible MAMS designs, that minimise a (weighted) loss function using a Bayesian optimality criterion – Supplemental Appendix B includes further details. We used the nstagebinopt and nstagebin Stata commands for this purpose.^11^ Both programmes allow the use of Dunnett’s correction for multiple testing.^12^

The maximum sample size remains too large for the trial to complete in a timely manner. To reduce this further, without compromising the statistical integrity of the design, two distinct stages are considered in the RED trial design. At the selection stage, a subset of research arms are selected to continue to the confirmatory stage with the control arm.

In MAMS selection designs, the selection of research arms can be made in multiple stages. The design can also allow for interim lack-of-benefit stopping rules as well as treatment selection. We will address these issues in section ‘Discussion’.

A distinctive feature of the MAMS selection design is that this pre-specified experimental arm selection is based on the ranks of the interim treatment effect estimates, whereas in the full MAMS design any experimental arm that passes the interim stopping rules has a chance to continue to the next stage. In the remainder of this article, we compare the operating characteristics of different MAMS selection designs against each other and the full MAMS, in the RED trial setting.

## Methods

In this section, we briefly describe the operating characteristics of MAMS selection designs – see Supplemental Appendix C for a formal and generic specification of the design and how it can be realised.

### Operating characteristics

#### Type I error rates when selecting experimental arms

Two measures of type I error in a multi-arm trial are the pairwise (PWER) and familywise (FWER) type I error rates.^8^ In the RED trial, the familywise error rate (FWER) was the measure of interest since the interventions in two of the research arms were similar. The control of the FWER means that the probability of recommending the selected treatment when it is ineffective should be at most 2.5% one-sided. We used simulations to calculate the FWER by generating the joint distribution of the test statistics at different stages with the underlying correlation structure. Supplemental Appendix C presents details on the calculation of the FWER.

#### Probability of correct selection

The probability of correct selection is the probability the most effective arm is selected at an interim stage – see Supplemental Appendix C and Kunz et al.^13^ for analytical derivations. In MAMS selection designs, it is desirable to have high probability of correct selection at the interim decision point since the overall power is bounded by this quantity. We used simulations to calculate it empirically by counting the average number of simulated trials which select the efficacious research arm at stage 1.

#### Overall power when selecting experimental arms

In the RED trial design, overall power is defined as the probability that the most effective arm is selected at the interim analysis and the primary null hypothesis is rejected for this arm at the final analysis – see Supplemental Appendix C and Kunz et al.^13^ for analytical derivations. We carried out simulations to calculate it empirically under different configurations of the underlying treatment effects. To be consistent, we present the scenario when one research arm has the target treatment effect in Results. The results for other scenarios are included in Supplemental Appendix F of the online Supplementary Material. They are summarised in section ‘Results’.

### Simulation study design

In this section, we describe the trial design parameters in our simulations based on the RED trial.

#### Trial design parameters

Table 1 presents the design parameters in our simulation studies – Supplemental Appendix D includes further details. We considered different timings of treatment selection in our simulations to investigate its impact on the operating characteristics of the design. This was done based on the control arm information times, that is, the proportion of total control arm patients in the selection stage. Given the low event rate in this trial, it is important to have a large enough sample size in the selection stage to decrease the uncertainty around the estimated treatment effects. For the sake of brevity, we will present the results for the following selection times: 11%, 17%, 21%, and 24% which correspond to the first-stage significance levels ( $\alpha_{1}$ ) of 0.70, 0.60, 0.55, and 0.50, respectively. The selection times considered provide a reasonable trade-off between accruing a sufficient sample size for treatment selection and control of the overall operating characteristics.

**Table 1.** Design parameters for MAMS selection designs used in simulations.

Design parameter ( I=D design)	MAMS selection design	
	Stage 1 (selection)	Stage 2
Primary outcome	MMS	MMS
No of research arms	3	1
Selection inf. time	11% or 17% or 21% or 24%	–
Sig. levels (one-sided), α	0.70 or 0.60 or 0.55 or 0.50	0.015
Design pairwise power	0.95	0.88
Ctrl. arm event rate	0.15	0.15
Treat. effect under $H_{0}$	0	0
Target treat. effect, $H_{1}$	θ:−0.05	θ:−0.05
Allocation ratio (C:E)	1:1 or 2:1 or 2:3 or 1:2	1:1 or 2:1

MAMS: multi-arm multi-stage; MMS: maternal mortality or invasive surgical procedures, (binary) primary outcome.

The same definitive (binary) outcome (MMS) is used at stage 1 for treatment selection.

The corresponding stage 1 (and maximum) sample sizes are presented in columns 4 and 5 of Tables 2 and 3. The above significance levels can also act as the lack-of-benefit stopping rule at the selection stage. We will address the design implications of such stopping boundaries in section ‘Discussion’. Finally, the design pairwise significance level for the selected research arm at stage 2 ( $\alpha_{2S}$ , where S stands for selected) is 0.015 (one-sided) in all experimental conditions. A grid search procedure was used to find $\alpha_{2S}$ (and the design pairwise power at stage 2) to ensure the control of both the FWER and overall power.

**Table 2.** The operating characteristics of different four-arm two-stage (MAMS) selection designs, I=D . scenario.

Design	AR (C:E)		Stage 1 SS (C:E)	Max. SS	Probability of correct Selection	Overall power	FWER
	Stage 1	Stage 2					
(i) Selection at 11% of information time							
1	1:1	1:1	109:109	2164	0.82	0.73	0.023
2	2:1	2:1	154:77	2218	0.77	0.67	0.023
(ii) Selection at 17% of information time							
1	1:1	1:1	168:168	2282	0.88	0.78	0.025
2	2:1	2:1	238:119	2302	0.83	0.72	0.026
(iii) Selection at 21% of information time							
1	1:1	1:1	201:201	2348	0.90	0.80	0.026
2	2:1	2:1	284:142	2348	0.85	0.75	0.027
vi) Selection at 24% of information time							
1	1:1	1:1	235:235	2416	0.92	0.82	0.027
2	2:1	2:1	333:167	2399	0.88	0.76	0.028

AR: allocation ratio; FWER: familywise type I error rate; SS: sample size.

The design (pairwise) power in all scenarios is 0.95 and 0.88 in stages 1 and 2, respectively. The design pairwise significance level for the selected arm at stage 2 analysis is $\alpha_{2S}=0.015$ (one-sided) in all scenarios. The number of simulations is 250,000 in each experimental condition.

**Table 3.** Comparison of the operating characteristics of the MAMS selection design with full MAMS and two-arm designs in terms of overall power and maximum sample size.

Design	AR (C:E)	Max. SS	Pr. corr. sel.^a^	Overall power	FWER
(i) MAMS selection designs					
(1) Selection at 11% inf. time	1:1	2164	0.82	0.73	0.023
(2) Selection at 17% inf. time	1:1	2282	0.88	0.78	0.025
(3) Selection at 21% inf. Time	1:1	2348	0.90	0.80	0.026
(4) Selection at 24% inf. time	1:1	2416	0.92	0.82	0.027
(ii) Other designs					
(1) Optimal (full) MAMS	2:1	3164	−	0.80^b^	0.025
(2) Two-arm trial	1:1	1366	−	0.80	0.025

AR. allocation ratio; FWER: familywise type I error rate; MAMS: multi-arm multi-stage; SS: sample size.

The design (pairwise) power in MAMS selection designs is 0.95 and 0.88 in stages 1 and 2, respectively. The design pairwise significance level at stage 2 is $\alpha_{2S}$ = 0:015 (one-sided) in all selection designs. The design pairwise power ( $\omega_{j}$ ) and significance levels ( $\alpha_{j}$ ) for the optimal MAMS design are (0.98, 0.80) and (0.26, 0.009), respectively. Supplemental Appendix B includes the Stata code for sample-size calculations.

a Probability of correct selection.

b The overall pairwise power in the optimal full MAMS design.

#### Allocation ratio

For a fixed-sample (one-stage) multi-arm trial, the optimal allocation ratio (C:E), that is, the one that minimises the sample size for a fixed power, is $\sqrt{K}$ . In designs with binary outcomes, this is locally optimal since the lower event rate in the experimental arms points to the optimal allocation of having slightly more in the experimental arms. However, in MAMS selection designs, the research arms are implicitly compared against each other at the selection stage. For this reason, it might be more efficient to randomise more individuals to the research arms during the selection stage. This decreases the uncertainty of the estimated risk from the research arms, which can increase the probability of correct selection and power of a MAMS selection design. For this reason, we considered different allocation ratios which are presented in Table 1. Note that the change in allocation ratio is pre-specified in all design scenarios, and is independent of stage 1 results. This was done under the assumption that there is no time trend in the study population, which may increase the risk of bias and may result in the loss of efficiency.^14^

#### Outcome measure for treatment selection

In a MAMS design, the use of an I outcome at the interim stages speeds up the weeding out of insufficiently promising treatments. As a result, recruitment to the unpromising arms will be discontinued much faster than otherwise. Choosing an appropriate I outcome is key to the success of the design.^2^

There are two main requirements for a suitable I outcome in this setting. First, ‘information’ on *I* should accrue at the same rate or faster rate than information for the D outcome, where information is defined as the inverse of the variance of the treatment effect estimator. The second assumption is that the I outcome is on the pathway between the treatments and the D outcome. If the null hypothesis is true for I , it must also hold for D . In this setting, the I outcome does not have to be a perfect or true surrogate outcome for the definitive outcome as defined by Prentice.^15^

However, in MAMS selection designs, the focus is on the selection of the best-performing arms rather than dropping them for the lack-of-benefit. For this reason, there needs to be a reasonable trial-level correlation between the treatment effect on the I outcome and the treatment effect on the D outcome in all pairwise comparisons.^16^ Otherwise, the best-performing treatment on the I outcome may not always be the most promising on the primary clinical outcome of the trial. We carried out simulation studies to investigate the impact of treatment selection based on an I outcome on the operating characteristics and maximum sample size of a MAMS selection design. The design specification for the I≠D setting is presented in Table 4.

**Table 4.** Design parameters for an MAMS ( I≠D ) selection design.

Design parameter ( I≠D design)	MAMS selection design, I≠D	
	Stage 1 (selection)	Stage 2
Outcome type	continuous	binary
Outcome^a^	log(VBL)	MMS
Correlation $\left(\rho_{\hat{\mu }\hat{\theta }}\right)$ ^b^	0.50	0.50
Significance level (1-sided)	0.025	0.021
Design pairwise power	0.90	0.88
Ctrl. arm outcome mean (SD)	2.32 (0.4)	0.15 (0.36)
Treatment effect under *H*_0_	0	0
Target treatment effect (*H*_1_)	*µ*: −0.2	*θ*: −0.05
Allocation ratio (C:E)	1:1	1:1
Sample size in each arm	85	890
Overall operating characteristics and maximum sample size Maximum sample size 1950 Probability of correct selection 0.98 Overall power 0.87 FWER^c^ 0.025		

FWER: familywise type I error rate; MAMS: multi-arm multi-stage; MMS: maternal mortality or invasive surgical procedures; SD: standard deviation; VBL: volume blood loss.

The continuous outcome is used at stage 1 for treatment selection, whereas the primary binary outcome is used in stage 2 analysis.

a MMS is the primary (D) outcome which is tested at the final stage. The log (VBL) is the intermediate (I) outcome used for treatment selection at stage1.

b Correlation between the *I* and *D* outcome measures, that is, $\rho_{\hat{\mu }\hat{\theta }}=\mathrm{corr}({\hat{\mu }}_{\mathrm{jk}},{\hat{\theta }}_{\mathrm{jk}})$ , where *j* = 1; 2 and *k* = 1; 2; 3, at a fixed time point, assumed to be constant and the same for all pairwise comparisons –*j* and *k* are the stage and comparison indicators.

c FWER is calculated under the global null hypothesis for both the *I* and *D* outcome measures.

## Results

In this section, we present the results of our empirical investigation to explore the operating characteristics of the MAMS selection design. We present the trial design settings, as well as the stage 1 (and maximum) sample sizes that are derived from these designs. The performance measures that quantify the operating characteristics of each design are presented in the last three columns in all tables.

### Simulation results

#### Selection of experimental arms based on the definitive (D) outcome

The simulation results are summarised in the last three columns of Tables 2 and 3. First, the results suggest that the probability of correct selection, power, and the FWER increase with the timing of treatment selection. For example, for the fixed 1:1 allocation ratio, the corresponding quantities are 0.82, 0.73, and 0.023, respectively, when the decision to select the best treatment is made at 11% information time. They increase to 0.88, 0.78, and 0.025, respectively, when the selection is carried out at 17% information time.

Second, our results indicate that randomising more patients to the research arms in the first stage increases the probability of correct selection and power. However, it also increases the overall type I error rate – that is, the last column of Table 1 in Supplemental Appendix E. The results suggest that a fixed allocation ratio of 1:1 is the best option when only one research arm is selected to continue to the next stage. Overall, treatment selection at 17% information time gives the smallest maximum sample size and highest power where the FWER controlled at 2.5% (one-sided), that is, design (ii-1) in Table 2.

Table 3 compares the maximum sample size and the operating characteristics of the MAMS selection designs with fixed 1:1 allocation ratio with those of the optimal full MAMS design. The chosen design with treatment selection at 17% information time (i-2) decreased the maximum sample size by 28% compared with that of the optimal full MAMS design with comparable operating characteristics. Its maximum sample size (2282) is even smaller than the expected sample sizes of the optimal MAMS design – that is, 2574 and 3151 under the null and alternative hypothesis, respectively. Note that in two-stage selection designs where only one arm is selected at stage 1, the maximum and expected sample size are the same under the non-binding lack-of-benefit stopping rules.

Finally, further simulations to explore the performance of the optimal full MAMS and the chosen MAMS selection design, that is, design (i-2) in Table 3, under different configurations of the underlying treatment effects indicate that the lowest power for the MAMS selection design is achieved when one arm has the target effect size and the underlying effect sizes in the other research arms is 50% of the target effect size – see Supplemental Appendix F. Note that any design will lose power under smaller effect sizes. For example, the power of a two-arm design reduces to 0.20 with an effect size of −0.025 from 0.80 when the effect size is −0.05 (see Table 2 in Supplemental Appendix F). Nonetheless, the overall power of the chosen MAMS selection design is only 4% lower at 0.74 in this case.

#### Selection of experimental arms based on an *I* outcome

Volume blood loss (VBL) is used as the outcome of interest in the early phase postpartum haemorrhage trials since the loss of large amounts of blood postpartum can lead to severe maternal morbidity and mortality. The VBL is an information-rich continuous outcome that follows lognormal distribution.^17^ We used past early phase trials in PPH to specify the selection stage design parameters in this new setting. Table 4 presents the design parameters, maximum sample size, and overall operating characteristics of the MAMS selection design when the log(VBL) is used for treatment selection at stage 1 – further details are included in Supplemental Appendix D.

While for the I=D design we compared the overall operating characteristics across various designs to find the best selection design, in the I≠D design our aim is to show how the correlation structure between the two treatment effects, that is, $\rho_{\hat{\mu }\hat{\theta }}=\mathrm{corr}(\hat{\mu },\hat{\theta })$ , as well as the underlying treatment effect on the I outcome can affect the overall operating characteristics of a selection design in this setting. $\rho_{\hat{\mu }\hat{\theta }}$ is the trial-level correlation between the treatment effect on I and the treatment effect on D , which is different from the patient-level correlation between the I and D outcomes.^16^ An estimate of $\rho_{\hat{\mu }\hat{\theta }}$ is required to calculate the overall operating characteristics of the MAMS ( I≠D ) selection design.^18^

At the time of the design, no information was available about $\rho_{\hat{\mu }\hat{\theta }}$ . In the FWER and power calculations in Table 4, we used a correlation of $\rho_{\hat{\mu }\hat{\theta }}=0.50$ . In our simulations, we assumed three values for $\rho_{\hat{\mu }\hat{\theta }}$ , {0.20,0.50,0.65} , to explore its impact on the operating characteristics of the design. We used a grid search procedure to choose the final stage significance level ( $\alpha_{2S}$ ) such that the overall FWER is at the pre-specified level of 2.5% (one-sided), that is, $\alpha_{2S}=0.021$ , and calculated the reported FWER in Table 4 under the global null hypothesis for both the I and D outcome measures. The maximum sample size of this MAMS ( I≠D ) selection design is about 15% lower with much higher overall power. However, this design is only robust under the assumption that there is a reasonable trial-level correlation between treatment effect on the VBL and the treatment effect on the MMS in all pairwise comparisons.^16^ This assumption does not hold in the RED trial setting. Unlike the other two devices, the STUT device has a different mechanism of action. It has been shown to be effective in previous pilot studies. However, it has a suction mechanism that may increase the average blood loss for some individuals if it is measured for short periods.

We conducted further simulations to explore the impact of the correlation structure between the I and D outcome measures ( $\rho_{\hat{\mu }\hat{\theta }}$ ), the variance of the I outcome, and the I outcome true treatment effect ( μ ) on the operating characteristics of this design. In simulations, we generated patient-level correlated outcome data and calculated the corresponding outcome measures and test statistics. To decrease the Monte Carlo error of the FWER, the simulations were run with 100,000 replications under the global null hypothesis, and 10,000 replications for power calculation.

The results are presented in Table 5 for two different values for $\rho_{\hat{\mu }\hat{\theta }}=(0.50,0.65)$ and two underlying effects for the I outcome measure in the selection stage. In one scenario, we simulated the I outcome under the target effect size of μ=−0.2 , that is, (i-1) in Table 5. We also simulated the I outcome under smaller effect size ( μ=−0.1 ) and a larger variance for the I outcome since the suction device might increase the variability in the VBL, (i-3) in Table 5. In all simulation scenarios, the true effect size for the definitive clinical outcome measure was the same, that is, θ=−0.05 .

**Table 5.** Comparison of the operating characteristics of the four-arm two-stage (MAMS) selection design presented in Table 4 under two different correlation structures between I and D outcome measures, $\rho_{\hat{\mu }\hat{\theta }}=\mathrm{corr}({\hat{\mu }}_{\mathrm{jk}},{\hat{\theta }}_{\mathrm{jk}})$ , and true treatment effect on the I outcome.

Scenario ( I≠D )	Stage 1 selection			Max. SS	Probability of correct selection	Overall power	FWER^a^
	*I* outcome	*I*-outcome true effect (SD)	Ctrl. arm SS (inf. time)				
(i) $\rho_{\hat{\mu }\hat{\theta }}=0.50$							
1	log(VBL)	−0.2 (0.40)	85 (10%)	1950	0.98	0.87	0.025
2	log(VBL)	−0.2 (0.80)	85 (10%)	1950	0.94	0.83	0.029
3	log(VBL)	−0.1 (0.40)	85 (10%)	1950	0.80	0.71	0.025
4	log(VBL)	−0.1 (0.80)	85 (10%)	1950	0.74	0.65	0.029
(ii) $\rho_{\hat{\mu }\hat{\theta }}=0.65$							
1	log(VBL)	−0.2 (0.40)	85 (10%)	1950	0.98	0.87	0.027
2	log(VBL)	−0.2 (0.80)	85 (10%)	1950	0.95	0.84	0.032
3	log(VBL)	−0.1 (0.40)	85 (10%)	1950	0.80	0.71	0.027
4	log(VBL)	−0.1 (0.80)	85 (10%)	1950	0.74	0.66	0.032

FWER: familywise type I error rate; SD: standard deviation; SS: sample size; VBL: volume blood loss.

The target effect size of −0.2 is assumed for the stage 1 sample size calculation with a standard deviation of 0.4 for the log volume blood loss. The true and target effect for the definitive ( D ) clinical outcome is the same in all scenarios, that is, θ=−0.05 . The design (pairwise) significance levels ( $\alpha_{1k},\alpha_{2S}$ ) and powers ( $\omega_{1k},\omega_{2S}$ ) are (0.025, 0.021) and (0.90, 0.88), respectively, in all experimental conditions. The number of replications is 100,000 (and 10,000) for the FWER (and power) calculations in all scenarios.

a FWER is calculated under the global null hypothesis for both the I and D outcome measures.

The simulation results in Table 5 indicate that the correlation between the I and D outcome measures has a negligible impact on the probability of correct selection and overall power. However, the FWER increases as the correlation between the outcome measures increases. We also carried out simulations under $\rho_{\hat{\mu }\hat{\theta }}=0.20$ ; the FWER and overall power were 0.023 and 0.86, respectively. Finally, the simulation results indicate that an increase in the variance of the log (VBL) will decrease the overall power on the D outcome measure, whereas it increases the overall FWER – that is, scenarios (i-2) and (ii-2) in Table 5.

## Discussion

In this article, we investigated pre-specified treatment selection under the Royston et al.’s MAMS framework. Motivated by the RED trial in refractory postpartum haemorrhage, we compared the operating characteristics and maximum sample size of MAMS selection designs with that of the optimal full MAMS design and two-arm trials. As demonstrated in the RED trial design and in our extensive simulation studies, MAMS selection designs can produce maximum sample size savings of up to 28% compared to the optimal (full) MAMS design and by about 44% compared with 3 two-arm trials.

In MAMS selection designs, the primary aim is to select the most promising treatments with high probability of correct selection where strong control of the error rates is required in the phase III setting. The probability of correct selection and overall power are driven by the underlying treatment effects, timing of selection, and the number of comparisons. Our findings suggest that treatment selection at about one-fifth of the control arm information time gives the smallest maximum sample size when only one research arm is selected to continue to the next stage. The optimal allocation ratio is also 1:1 in this case, both at the selection and confirmatory stages. This also reduces the risk of bias and loss of efficiency when time trends are present.^14^

In this study, we selected the best research arm that had the largest treatment effect. However, the selection of research arms can be made based on a combination of efficacy and safety data. The incorporation of other (safety) outcomes to determine selection will not increase the type I error rate of a MAMS selection design since the FWER is maximised by selecting the best-performing arm.^19^ However, the overall power may be adversely affected since not selecting the best-performing arm can lead to a conservative procedure.^20^ Furthermore, in MAMS selection designs with several research arms, more than one research arm can be selected at the selection stage to increase power. This needs to be pre-specified; otherwise, the overall type I error rate will increase beyond the nominal value. Or the selection of promising research arms can be done in several stages – this is an area for further research.

In some studies, independent monitoring committees and funding agencies might require interim stopping boundaries and clear guidelines as to when the trial should be terminated. This can be achieved by including lack-of-benefit stopping rules as part of the selection criteria, or by introducing a further interim lack-of-benefit analysis for the selected treatment arm(s). Our simulation results, presented in Supplemental Appendix G of the online Supplementary Material, indicate that in the RED trial design the impact of such a stopping rule on the overall power is minimal, that is, less than 2% reduction. In principle, the MAMS selection design can also allow for interim stopping boundaries for overwhelming efficacy.^3^ This can potentially increase the overall type I error rate, which can be corrected using simulations.

Moreover, we explored treatment selection based on the I outcome of the VBL in the RED trial. This outcome was not used for treatment selection since one device has a different mechanism of action from that of the other devices. We showed that sample size can be reduced further by using an I outcome for treatment selection. In this setting, the research arms can only be robustly selected if there is a reasonable trial-level correlation between the treatment effect on the I outcome and the treatment effect on the D outcome in all pairwise comparisons.^16^ Nonetheless, even in this case the reduction in the maximum sample size would have been moderate (around 15%) in the RED trial. When selecting based on the treatment effect of the I outcome, it is recommended to have a number of selection stages to reduce the number of experimental arms gradually, for example, reducing one arm only at each stage, if the I outcome data suggest that several treatments perform similarly and better than the control.^18^

There are further design considerations when selecting based on the treatment effect of the I outcome. First, an estimate of the correlation between the I and D outcome measures ( ρ ) is required to calculate the operating characteristics of the selection design. When the I and D outcomes are of different distributions, the correlation between the corresponding test statistics is unlikely to be high. In several cases, reported values were between 0.10 and 0.40.^21^ In studies that no such information is available, such as the RED trial, the operating characteristics of the design can be calculated based on an initial estimate for the correlation. The initial value should be updated as trial data is accumulated. Then, the operating characteristics of the design should be re-calculated using the upper bound for the estimated correlation. To control the FWER, the final stage critical value might have to be adjusted which can increase the maximum sample size.

Finally, in designs with long-term outcome measures, similar simulation studies to those presented in this article should be performed to assess the operating characteristics of the selection design as well as the efficiency gains in terms of the maximum sample size and trial timelines.

## Conclusion

MAMS selection designs can be more suitable than the full MAMS design in certain circumstances, particularly in resource-limited settings where a number of candidate research treatments are available. Table 6 summarises the pros and cons of each approach and situations when they might be used. The pros and cons of each approach would depend on the clinical setting as well as the types of primary and intermediate outcomes used for the trial design.

**Table 6.** The MAMS selection and full MAMS design: summary of their pros and cons and situations when they might be used.

MAMS selection designs	Full MAMS designs^a^
Suitable when there is likely to be clear and strict limit on the number of individuals that can be recruited, and/or the funds available to support the trial, and/or when the timeline for the trial is specifically restricted. These constraints can often mean that not all research treatments can be continued to accrue sufficient individuals for the primary analysis of the primary outcome measure.	Suitable when there is sufficient individuals and funding such that accrual to all research arms is possible to undertake the primary analysis of the primary outcome measure.
More suited when the research treatments are related in some way, for example, different doses or durations.	Preferable when the research arms testing interventions which are biologically distinct from each other.
Have smaller maximum sample size and associated costs.	Can have smaller expected sample size in designs with an intermediate outcome.
The efficiency gains depend on the number of research treatments: maximum sample size and overall type I and II error rates may increase with increasing number of research arms.	Can be more efficient, particularly when a suitable intermediate outcome is used for the interim lack-of-benefit analysis.
Interim changes to the pre-specified design parameters can adversely affect its operating characteristics and efficiency.	If more than one research arm is found to be effective then in this design it may be possible to consider adding the interventions in the research arms, as they will typically have different modes of action if the MAMS design is being used.

Non-exhaustive list; the advantages of each approach may also depend on the clinical setting and trial outcomes. MAMS: multi-arm multi-stage.

a See Royston et al.^1^ for MAMS design; see also Bratton et al.^22^ for the extension to binary outcomes.

## Supplemental Material

sj-pdf-1-ctj-10.1177_17407745221136527 – Supplemental material for Treatment selection in multi-arm multi-stage designs: With application to a postpartum haemorrhage trial Supplemental material, sj-pdf-1-ctj-10.1177_17407745221136527 for Treatment selection in multi-arm multi-stage designs: With application to a postpartum haemorrhage trial by Babak Choodari-Oskooei, Soe Soe Thwin, Alexandra Blenkinsop, Mariana Widmer, Fernando Althabe and Mahesh KB Parmar in Clinical Trials
