# Supplementary material for: Treatment selection in multi-arm multi-stage designs: With application to a postpartum haemorrhage trial
Source: Clin Trials. 2023 Jan 17;20(1):71–80. doi: 10.1177/17407745221136527 (PMC9940121; doi:10.1177/17407745221136527)
Supplement: sj-pdf-1-ctj-10.1177_17407745221136527 – Supplemental material for Treatment selection in multi-arm multi-stage designs: With application to a postpartum haemorrhage trial [file sj-pdf-1-ctj-10.1177_17407745221136527.pdf]

Appendix A: Steps to design a MAMS trial

The following steps should be taken to design a MAMS trial with both lack-of-benefit and efficacy stopping boundaries see - see Choodari-Oskooei et al.[1] for further guidelines:

1. Choose the number of experimental (E) arms, K, and stages, J.
2. Choose the definitive D outcome, and (optionally) I outcome.
3. Choose the null values for θ - e.g. the (log) hazard ratios on the intermediate (θ_I^0) and definitive (θ_D^0) outcomes.
4. Choose the minimum clinically relevant target treatment effect size, e.g. in the time-to-event setting the (log) hazard ratio on the intermediate (θ_I^1) and definitive (θ_D^1) outcomes.
5. Choose the control arm event rate (median survival) in trials with binary (survival) outcome.
6. Choose the allocation ratio A (E:C), the number of patients allocated to each experimental arm for every patient allocated to the control arm. For a fixed-sample (1-stage) multi-arm trial, the optimal allocation ratio (i.e. the one that minimizes the sample size for a fixed power) is approximately $A = 1/\sqrt{K}$.
7. In $I \neq D$ designs, choose the correlation between the estimated treatment effects for the I and D outcomes. An estimate of the correlation can be obtained by bootstrapping relevant existing trial data.
8. Choose the accrual rate per stage to calculate the trials timelines .
9. Choose a one-sided significance level for lack-of-benefit and the target power for each stage (α_{jk}, ω_{jk}). The chosen values for α_{jk} and ω_{jk} are used to calculate the required sample sizes for each stage.
10. Choose whether to allow early stopping for overwhelming efficacy on the primary (D) outcome. If yes, choose an appropriate efficacy stopping boundary α_{Ej} on the D-outcome measure for each stage $1, ..., J$, where $\alpha_{EJ} = \alpha_J$. Possible choices are Haybittle-Peto or O'Brien-Fleming stopping boundaries used in group sequential designs, or one based on α-spending functions (Blenkinsop et al. 2019[2]).
11. Given the above design parameters, calculate the number of control and experimental arm (effective) samples sizes required to trigger each analysis and the operating characteristics of the design, i.e. n_{jk} in trials with continuous and binary outcomes and e_{jk} in trials with time-to-event outcomes, as well as the overall type I error rate and power. If the desired (pre-specified) overall type I error rate and power have not been maintained, for instance if the overall pairwise power is smaller than the pre-specified value, steps 9-11 should be repeated until success. Or, if the overall type I error rate is larger than the pre-specified value, one can choose a more stringent (lower) design alpha for the final stage, α_J, and repeat steps 9-11 until the desired overall type I error rate is achieved.

Appendix B: optimal full MAMS design for the RED trial

In this Appendix, we give the Stata code to find the optimal full MAMS design for the RED trial. The `nstagebinopt` program finds a class of efficient 'admissible' designs based on a Bayesian optimality criteria using a systematic search procedure. The `nstagebin` command calculates the stagewise sample sizes, trial timelines, and the overall operating characteristics of MAMS design with binary outcomes. Both programs allow the use of Dunnett's correction to account for multiple testing[3].

In multi-arm trials, `nstagebinopt` outputs the stagewise operating characteristics and expected sample sizes under the global null and alternative hypotheses, i.e. $E(N|H_0)$ and $E(N|H_1)$, for each admissible J-stage design which minimises the Bayesian optimality criteria proposed by[4], i.e. $L(q) = qE(N|H_1) + (1-q)E(N|H_0)$ for some $q \in [0, 1]$. The program can also save this information in a Stata dataset by specifying the `save()` option and can produce a plot of $E(N|H_0)$ versus $E(N|H_1)$ by choosing the `plot` option. Each admissible design can then be entered into the `nstagebin` program to explore them in more details - both code and output are included below.

In the RED trial, the control arm MMS rate was assumed to be 15%, i.e. specified using `ctrlp(0.15)` option in both programs. The trial is powered to detect a target MMS rate of 10% in each of the experimental arms, an absolute reduction of $\theta = 5\%$, `theta1(-0.05)`, and relative reduction of 33.3%. Patients are allocated to the control arm with a 2:1 ratio, `aratio(0.5)`, to increase power for each of the pairwise comparisons. In both stages, the accrual rate was (on average) assumed to be 500 patients year - bearing in mind that this only affects the stage durations and the trial timelines.

The output from `nstagebinopt` is shown below for the RED MAMS trial with the (one-sided) FWER of 0.025, `alpha(0.025)`, and the pairwise power of 0.80, `power(0.80)`. Note that the FWER is calculated using simulations in both `nstagebin` and `nstagebinopt` commands. For this reason, both programs calculate (and present) the corresponding Monte Carlo error (SE) using the formula: $\sqrt{\frac{FWER\times(1-FWER)}{N}}$, where $FWER$ is the calculated overall familywise type I error rate and N is the number of simulations. The range of values of q ('q-range') for which each design minimises the loss function are also presented. Minimax designs (admissible for $q = 1$) use a high power in the intermediate stages so that the lowest possible power is chosen in the final stage, thus reducing the maximum sample size - see design with $q \in [0.90, 0.98]$ in the `nstagebinopt` output. The stagewise powers in the intermediate and final stages then balance out as q decreases (i.e. as $E(N|H_0)$ becomes more of a factor in choosing a design). The general pattern observed in the output, is that as the expected sample size of the admissible designs increases under the global alternative hypothesis, $E(N|H1)$, the expected sample size under the global null hypothesis, $E(N|H0)$, decreases.

The results indicate that the design which is admissible for $q \in [0.90, 0.98]$ has an expected sample size of 3151 patients which is just 2 patients higher than the minimax design with 3149 patients. However, this admissible design has a much smaller $E(N|H_0)$ than that of the minimax design. The design which is admissible for $q \in [0.41, 0.89]$ has an expected sample size of 2035 under global null hypothesis, $E(N|H0)$, but the maximum sample size for this design is 3310 which is higher than the one below, i.e. admissible for $q \in [0.90, 0.98]$, with the maximum sample size of 3164. Overall, the design that is admissible for $q \in [0.90, 0.98]$ was selected as the preferred choice - since our focus is reducing the maximum sample size and this design provides one of the lowest expected sample size under the global alternative hypothesis. So, the chosen stagewise significance levels and powers were used in the `nstagebin` command for sample size calculations.

Next, the chosen design stagewise power and significance levels obtained from the `nstagebinopt` output were used in the `nstagebin` command to calculate the required sample size for the optimal full MAMS design for the RED trial. Most of the design parameters have been defined in the previous section. The selected significance levels are 0.26 and 0.009 - i.e. `alpha(0.26 0.009)`. The selected design stagewise powers (ω_{jk}) are 98% and 80%, respectively for each of the two stages in all 3 pairwise comparisons - `power(0.98 0.80)`. These stagewise design parameters ensure an overall familywise type I error rate (FWER) of 2.5% (one-sided) and a pairwise power of 80%.

```
nstagebinopt, nstage(2) arms(4) alpha(0.025) power(0.80) ///
                theta0(0) theta1(-0.05) ctrlp(0.15) accrate(500 500) aratio(0.5) fwer

 Finding set of feasible designs...
 Calculating expected sample sizes...
 Finding set of admissible designs...
--------------------------------------------------------------------------
    q-range    Stage     Sig.    Power   Alloc.  E(N|H0)  E(N|H3)     FWER
                         level            ratio                       (SE)
--------------------------------------------------------------------------
[0.00,0.24]        1      0.22     0.88     0.50     1929     3403   0.0253
                   2     0.011     0.87                             (0.0003)
--------------------------------------------------------------------------
[0.25,0.40]        1      0.15     0.88     0.50     1949     3340   0.0251
                   2     0.011     0.86                             (0.0003)
--------------------------------------------------------------------------
[0.41,0.89]        1      0.23     0.92     0.50     2035     3216   0.0251
                   2     0.010     0.83                             (0.0003)
--------------------------------------------------------------------------
[0.90,0.98]        1      0.26     0.98     0.50     2574     3151   0.0254
                   2     0.009     0.80                             (0.0003)
--------------------------------------------------------------------------
[0.99,1.00]        1      0.12     0.96     0.50     2730     3149   0.0245
                   2     0.009     0.80                             (0.0003)
--------------------------------------------------------------------------
 Note: each design minimises the loss function (1-q)E(N|H0)+qE(N|H1) for values
       of q specified in q_range. H1 is the hypothesis that all of the experimental
       arms are effective.

nstagebin, nstage(2) arms(4 4) alpha(0.26 0.009) power(0.98 0.80) theta0(0)  ///
theta1(-0.05) ctrlp(0.15) accrate(500 500) aratio(0.5) tunit(1) ess

n-stage trial design                 version 1.0.1, 17 Jul 2014
---------------------------------------------------------------
Sample size for a 4-arm 2-stage trial with binary outcome based
on Bratton et al. (2013) BMC Med Res Meth 13:139
---------------------------------------------------------------

Control arm event rate = 0.15

Operating characteristics
----------------------------------------------------------------------
           Alpha(1S)     Power  theta|H0  theta|H1   Length*     Time*
----------------------------------------------------------------------
Stage 1       0.2600     0.980     0.000    -0.050     4.475     4.475
Stage 2       0.0090     0.800     0.000    -0.050     1.850     6.325
Pairwise      0.0089     0.800                                   6.325
FWER (SE)     0.0253   (0.0003)
----------------------------------------------------------------------
 *  Length (duration of each stage) is expressed in year periods

Cumulative sample sizes per arm per stage
                       ---------Stage 1---------  ---------Stage 2---------
                       Overall  Control   Exper.  Overall  Control   Exper.
---------------------------------------------------------------------------
Number of active arms        4        1        3        4        1        3
Accrual rate*            500.0    200.0    300.0    500.0    200.0    300.0
Active arms
Patients for analysis     2239      895      448     3164     1265      633
Patients recruited**      2239      895      448     3164     1265      633
All arms
Patients recruited**      2239                       3164
---------------------------------------------------------------------------
 *  Accrual rates are specified in number of patients per year
 ** Accounts for loss-to-follow-up rate and includes those recruited during follow-up periods

Expected sample size | 0 effective arms = 2573
Expected sample size | 3 effective arms = 3151
```

Appendix C: Design specification in full MAMS and MAMS selection designs

Design specification in the RED trial

For a MAMS trial with K research arms and J stages, let π_k and π_0 be the probability of developing the (binary) outcome of interest at each stage in experimental arm k and the control arm, respectively. For simplicity in notation, we outline the design specification for the case where the same definitive (D) outcome is monitored throughout the trial, i.e. *I=D* design.

The treatment effect is the difference in risks, i.e. a reduction in an unfavourable event rate, and is being measured by $\theta_{jk} = \pi_{jk} - \pi_{j0}$ where $j = 1, \ldots, J$ and $k = 1, \ldots, K$. For simplicity, since we assume that all K pairwise comparisons have the same design parameters, i.e. design stagewise significance level (α_j) and power (ω_j), we remove the subscript k from the notations of design parameters. In line with the RED trial, we also assume $K = 3$ and $J = 2$ in all notations.

Without loss of generality, assume that a negative value of θ_{jk} indicates a beneficial effect of treatment k. In full MAMS trials with 3 experimental arms, a set of 3 null hypotheses are tested at each stage j: $H^0_{jk} : \pi_{j0} = \pi_{jk},\ \ j = 1, 2,\ \ k = 1, 2, 3$. For example, there are 6 sets of null and alternative hypotheses in a full MAMS design with 2 stages and 3 pairwise comparisons. In the MAMS selection design, the number of hypotheses depend on the selection rule, that is the maximum number of research arms to be selected at each stage. For example, in the 2-stage RED trial where only one research arm is selected at stage 1 interim, the primary null hypothesis tested at final analysis is $H_0 : \pi_{20} = \pi_{2S}$, where $S =$ selected arm. The design can also include interim stage lack-of-benefit analysis. We first present the two-stage selection design in this section. In the next section, we present the design specification for MAMS selection designs with both interim lack-of-benefit stopping boundaries and treatment selection.

Let Z_{jk} be the Z-test statistic comparing experimental arm k against the control arm at stage j where Z_{jk} follows a standard normal distribution, $Z_{jk} \sim N(0, 1)$ under the null hypothesis. Note that all the cumulative data from previous stages are used in the calculations of each Z-test statistic. In other words, the pairwise analyses at each stage includes all the individuals that were included in the analyses of previous stages. Ignoring selection, the joint distribution of the Z-test statistics therefore follows a multivariate normal distribution $MVN(\theta\sqrt{V}, \Sigma)$, where θ and V are the 2×3 matrices of the mean treatment effects and the corresponding Fisher's (observed) information ($V_{jk} = 1/\mathrm{Var}(\hat{\theta}_{jk})$), and Σ denotes the correlation matrix between the 2×3 test statistics. When there is prespecified treatment selection at stage 1, the three test statistics (Z_{11}, Z_{12}, Z_{13}) are ranked in order of size to determine which should proceed to the subsequent stage. The research arm with the highest rank is selected to continue to the next stage, and the other research arms cease recruitment and are discontinued. At the final analysis, the treatment effect on the primary outcome is estimated (with the corresponding p-values) for the selected arm (S) and includes all the individuals that were recruited in the selection stage for the control arm and the selected arm. As a result, one of two conclusions can be made:

- If $p_{2S} \leq \alpha_{2S}$, reject the null hypothesis corresponding to the definitive outcome and claim efficacy,
- If $p_{2S} \geq \alpha_{2S}$, the corresponding null hypothesis cannot be rejected at α_{2S} level,

where α_{2S} is the final stage significance level for the selected arm which is determined using numerical integration or simulations.

Design specification for MAMS selection designs with both treatment selection and lack-of-benefit interim analysis

In this section, we present the design specification for MAMS selection designs where the same primary outcome is used at interim analysis for treatment selection and lack-of-benefit analysis, i.e. $I = D$ selection designs. For a MAMS trial with K research arms and J stages, let π_k and π_0 to be the rate of the outcome at each stage in experimental arm k and the control arm, respectively. This may be the event rate for binary outcomes, or hazard rate for survival outcomes, for example. These rates do not differ by stage since it is assumed the same outcome measure is used throughout the trial (i.e. *I=D*). The treatment effect being measured is defined by $\theta_k = \pi_k - \pi_0$. Where a trial is seeking to identify a reduction in the outcome measure compared to the control arm, such as the hazard ratio in time-to-event outcomes, the null and alternative hypotheses for the risk difference at stage j for pairwise comparison k ($j = 1, \ldots, J$ and $k = 1, \ldots, K$) are defined by the following:

$$H_0 : \theta_{jk} \geq 0$$
$$H_1 : \theta_{jk} < 0$$

The direction of the hypotheses can be reversed if a trial is seeking an increase in the outcome measure compared to the control arm.

At each interim stage, the significance levels $\alpha_1, \ldots, \alpha_{J-1}$ are chosen for testing each pairwise comparison for lack-of-benefit. Let's assume that $L = (l_1, \ldots, l_{J-1})$ is the lower threshold for lack-of-benefit on the z-scale, determined by the chosen αs. A pre-specified selection rule is also defined by $S = (s_1 : \ldots : s_{J-1})$, where s_j is the maximum number of research arms to be selected at the end of stage j. The selection rule can also be written as $K : s_1 : s_2 : \ldots : s_{J-1}$ to reflect notation by others.[5] Note fewer arms may be selected if not all s_j arms pass the lack-of-benefit threshold. s_{J-1} can be greater than one, since the design allows for more than one null hypothesis may be rejected at the end of the trial, should several arms be found to demonstrate efficacy. Given the selection rule, the final stage significance level α_J is determined using numerical integration or simulations to ensure that the overall type I error rate (FWER) is controlled at the pre-specified level.

At each interim analysis, the test statistics $(Z_{j1}, \cdots, Z_{jk})$ are ranked in order of size, denoted by vector $\psi_j = (\psi_{j1}, \cdots, \psi_{jK})$, with the rank of arm k at stage j given by ψ_{jk}.

At each interim analysis, a decision based on two selection mechanisms is used to determine which should proceed to the subsequent stage:

- If $\psi_{jk} \leq s_j \bigcap Z_{jk} < l_j$, where $j = 1, \ldots, J-1$, then research arm k continues to the next stage
- If $\psi_{jk} > s_j \bigcup Z_{jk} > lj$, where $j = 1, \ldots, J-1$, then research arm k is dropped and ceases recruitment

The incorporation of other outcomes to determine selection, resulting in research arms which are not the best performing being selected, will not adversely impact the type I error rate, since it is maximised by selecting the best performing arm.[6] Therefore, whilst the number of arms to be selected must be pre-determined, it is not bound to the assumption that the selection must be based on the test statistic alone. However, the power may be adversely affected, since not selecting the best performing arm can lead to a conservative procedure.[7]

At the final analysis, the test statistics for each pairwise comparison for the remaining research arms are compared to the critical value for assessing efficacy, l_J, which is determined by α_J:

- If $Z_{Jk} > l_J$, the test is unable to reject H_0 at level α_J
- If $Z_{Jk} \leq l_J$, reject H_0 at level α_J and conclude efficacy for research arm k.

Operating characteristics

The key operating characteristics in a MAMS selection design are the overall probability of type I error rate (FWER), power and the probability of correct selection.

Type I error rates when selecting experimental arms

Two measures of type I error in a multi-arm trial are the pairwise (PWER) and familywise (FWER) type I error rates[8]. In the RED trial, the familywise error rate (FWER) was the type I error measure of interest since the interventions in two of the research arms were similar. Therefore, we wish to test the global null hypothesis that there is no difference between the treatment arms when one experimental arm is selected at stage 1. Let's denote S_k as the treatment that is selected at stage 1 and its treatment effect becomes statistically significant at stage 2. Then, the control of the FWER means that the probability of recommending the selected treatment when it is in fact ineffective, i.e. $\theta_{2k} \geq 0$, to be at most 2.5% one-sided. That is,

$$\Pr\{\bigcup_{k:\theta_{2k}\geq 0}(S_k \text{ recommended})\} \leq 0.025.$$

In the full MAMS setting, the Dunnett probability[3] can be used to calculate the FWER under the global null hypothesis assuming all promising arms are selected[2]. For MAMS selection designs, Stallard et al. developed analytical derivations for the type I and II error rates of two-stage designs - which was later extended to multi-stage setting[9 5]. It has been shown that the overall type I error rate of full MAMS design provides an upper bound for the MAMS selection design. In other words, full MAMS can be considered as a selection design where all research arms are available for selection if they passed previous stages. We used simulations to calculate the FWER. Analytical formulas can be found in[9,10] for designs when only one arm is selected and in[5,11] when multiple arms are selected to continue to the subsequent stages.

Probability of correct selection

The probability of correct selection is one of the key parameters in the MAMS selection design. It is the probability that the most effective arm is selected at an interim stage - see[12] for analytical derivations. In trials where only one research arm is selected at the interim stage, this probability is generally calculated under the assumption that one research arm is effective under the target effect size and the remaining arms are under the null. This probability can also be calculated under other hypotheses where, for example, multiple arms have the same treatment effect in which case the measure of interest would be the probability of any effective arm being selected. In a MAMS selection design, it is desirable to have high probability of correct selection at the interim decision points since the overall power is bounded by this quantity. It can be calculated analytically for a two-stage design. We used simulations to calculate it empirically by counting the average number of simulated trials which select the efficacious research arm at stage 1.

Power when selecting experimental arms

The simplest (and perhaps most useful) measure of power in a MAMS trial is the pairwise power for the comparison of each experimental arm k against control. However, two other types of power become relevant in the MAMS selection designs: any-pair and all-pair powers. Any-pair power (which corresponds to the definition of the FWER) is the probability of showing a statistically significant effect under the targeted effects for at least one comparison, all-pairs power is the probability of showing a statistically significant effect under the targeted effects for all the selected pairs. To calculate the overall power of the RED trial, data for arm k were generated under the target treatment effect, and the remaining arms were generated under the null (i.e. the remaining arms were ineffective). The three measures of power calculated are equal in this setting, and are defined as the probability of rejecting the effective research arm at the final analysis, conditional on its selection at the interim analysis. This approach to define pairwise power in a multi-arm setting with selection has been adopted by others[13].

Appendix D: Data generating mechanism in our simulation studies

The simulations aimed to assess the operating characteristics (e.g. FWER, probability of correct selection and overall power) of different MAMS selection designs under different scenarios. We recreated patient-level data aiming to replicate the proposed trial designs. Below, we outline further details

on simulations studies presented in the main body of the article.

$I = D$ designs

Trial designs for the $I = D$ scenarios presented in Table 1

The target effect size is assumed as 5% reduction in the control arm event rate of 15%, i.e. $\theta = -0.05$. To calculate stagewise sample sizes, we need to choose the stagewise design operating characteristics and allocation ratio. In MAMS selection designs, it is important to give effective arms a stronger chance of passing the selection stage, thus allowing more data to be collected on them. For this reason, the power in the selection stage should ideally be much higher than the final stage power. For this reason, design pairwise power of 0.95 was chosen for stage 1 sample size calculations in all pairwise comparisons. For stage 2, design pairwise power of 0.88 was chosen using a search procedure for the research arm that reaches the final stage to ensure high overall power over the two stages.

Data generating mechanism

Given design stagewise power and significance levels in the RED trial (see next section on how to choose them and Table 1 in the main text), the stagewise sample sizes were calculated using the `nstagebin` Stata command - code is presented in Appendix B. The primary binary outcome was generated for each observation in the sample assuming the following Bernoulli distributions for the control and experimental arms: $X \overset{\text{iid}}{\sim} Ber(\pi_0)$ in the control arm and $X \overset{\text{iid}}{\sim} Ber(\pi_k)$ in the experimental arm k, with $\pi_0 = \pi_k = 0$ under the global null hypothesis. Then, the observed number of responses in the control and each of the experimental arms follow binomial distributions with parameters: $Y_{j0} \sim Bin(n_{j0}, \pi_0)$ and $Y_{jk} \sim Bin(n_{jk}, \pi_k)$, respectively, where n_{jk} denotes the number of patients recruited to arm k between stages 1 and 2, and π_0, π_k the event rates in the control and research arms. Trial level data was generated for the each of the 2 stages under the binomial distribution. At stage 2, the data was added to the previous stage to induce the correlation between treatment effect estimates at stage 1 and 2 of the same pairwise comparisons. Correlation between different pairwise comparisons was also induced through the use of the shared control arm in calculating test statistics of each treatment comparison. The data generating mechanism was validated by checking the empirical distribution of different test statistics as well as verifying the empirical correlation against the expected theoretical values.

$I \neq D$ designs

Trial design for the $I \neq D$ scenario presented in Table 4

We used past early phase trials in PPH to specify the selection stage design parameters in this new setting. We agree that there's always an element of subjectivity in choosing these parameters. Nevertheless, the main objective of this section to show the implications of treatment selection based on an I outcome rather than finding the optimal design parameters. To calculate stage 1 sample size, the following design parameters should be specified for each comparison: stage 1 pairwise significance level and power; target effect size for the mean difference in log(VBL), i.e. $\mu_{1k} = \bar{x}_{1k} - \bar{x}_{10}$, which is assumed to be the same for all 3 experimental arms $k = 1, 2, 3$; and the allocation ratio. Althabe et al. used a design power of 0.80 with a significance level of 0.025 (one-sided) to power an early phase PPH trial with the continuous VBL as the primary outcome[14]. In the MAMS setting, it is important to choose higher power at interim stages. For this reason, a higher design pairwise power of 0.90 was chosen for stage 1 with the same significance level of 0.025 (one-sided). We could have chosen the same pairwise design power of 0.95 for stage 1, as we did for the selection based on the primary binary outcome. But, this would have increased the required sample size by 22% for the selection stage. Furthermore, from past trials we estimated the mean and standard deviation (SD) of the log(VBL) post randomisation for the control arm: mean (SD) of log(VBL) = 2.32 (0.4). Finally, a target effect size of 0.2 reduction in the mean log(VBL) was assumed to calculate the selection stage sample size - i.e. 0.5 standard deviation reduction in the mean of log(VBL). This resulted in a sample size of 85 per arm - with the same allocation ratio of 1:1 for all research arms.

Data generating mechanism in $I \neq D$ designs presented in Table 5

We generated two random correlated outcomes, one representing the stage-1 (selection stage) outcome (log volume blood loss), and the other the stage 2 (final analysis) outcome (maternal mortality of invasive surgical procedures). We drew correlated non-normally distributed outcomes by applying the following approach. First, we drew two random correlated values from a bivariate normal distribution. Then, we converted the normal draw to correlated uniforms, by applying the cumulative normal distribution function. Finally, we converted the uniform draws to the desired normal, log(VBL), and binary outcomes, by applying the inverse of the respective cumulative distribution functions.

Appendix E: Simulation results for other allocation ratios

Table 1 presents the simulation results for the adaptive allocation ratios in different designs. The operating characteristics of the designs calculated from the simulations are presented in the last three columns of the table. Overall, the results suggest that changing the allocation ratio after the selection stage do not result in higher efficiency in terms of lower (overall) type I and II error rates. Randomising more individuals to the experimental arms at the selection stage increases the probability of correct selection and overall power. But, this comes at a price with larger overall type I error rate with slightly larger maximum sample size.

Design	AR (C:E)		Stage 1	Max. SS	Pr. of correct	Overall	FWER
	Stage 1	Stage 2	SS (C / E)		selection	power	
i) Design scenario, $\alpha_1 = 0.70$ (i.e. selection at 11% inf. time for designs 1 and 2):							
1	$1:1$	$1:1$	109 / 109	2164	0.82	0.73	0.023
2	$2:1$	$2:1$	154 / 77	2218	0.77	0.67	0.023
3	$2:1$	$1:1$	154 / 77	2091	0.77	0.68	0.022
4	$2:3$	$1:1$	94 / 141	2239	0.85	0.76	0.024
5	$1:2$	$1:1$	87 / 174	2313	0.88	0.79	0.024
ii) Design scenario, $\alpha_1 = 0.60$ (i.e. selection at 17% inf. time for designs 1 and 2):							
1	$1:1$	$1:1$	168 / 168	2282	0.88	0.78	0.025
2	$2:1$	$2:1$	238 / 119	2302	0.83	0.72	0.026
3	$2:1$	$1:1$	238 / 119	2171	0.83	0.74	0.024
4	$2:3$	$1:1$	145 / 218	2399	0.91	0.82	0.026
5	$1:2$	$1:1$	134 / 268	2514	0.94	0.84	0.027
iii) Design scenario, $\alpha_1 = 0.55$ (i.e. selection at 21% inf. time for designs 1 and 2):							
1	$1:1$	$1:1$	201 / 201	2348	0.90	0.80	0.026
2	$2:1$	$2:1$	284 / 142	2348	0.85	0.75	0.027
3	$2:1$	$1:1$	284 / 142	2216	0.85	0.76	0.024
4	$2:3$	$1:1$	173 / 260	2485	0.93	0.84	0.026
5	$1:2$	$1:1$	159 / 318	2623	0.95	0.85	0.028

Table 1. The operating characteristics of different 4-arm 2-stage (MAMS) selection designs, $I = D$ scenario. The design (pairwise) power in all scenarios are 0.95 and 0.88 in stages 1 and 2, respectively. The design pairwise significance level for the selected arm at stage 2 analysis is $\alpha_{2S} = 0.015$ (one-sided) in all scenarios. Number of simulations is 250,000 in each experimental condition. AR, allocation ratio; SS, sample size; FWER, familywise type I error rate, calculated under the global null hypothesis.

Appendix F: The operating characteristics of the full MAMS and MAMS selection designs under different configurations of treatment effects and selection rules

F1: Power of the 4-arm 2-stage optimal full MAMS design

Scenario	No of effective arms	Assumed effect sizes $(\theta_1, \theta_2, \theta_3)$	Max. SS	Pairwise power			Any-pair power❀	All-pair power✻	FWER
				Arm 1	Arm 2	Arm 3			
1	1	$-0.05, 0, 0$	3164	0.80	–	–	0.80	0.80	0.025
2	2	$-0.05, -0.025, 0$	3164	0.80	0.20	–	0.82	0.18	0.025
3	2	$-0.05, -0.05, 0$	3164	0.80	0.80	–	0.93	0.67	0.025
4	3	$-0.05, -0.025, -0.025$	3164	0.80	0.20	0.20	0.83	0.07	0.025
5	3	$-0.05, -0.05, -0.025$	3164	0.80	0.80	0.20	0.93	0.17	0.025
6	3	$-0.05, -0.05, -0.05$	3164	0.80	0.80	0.80	0.97	0.58	0.025

Table 2. The operating characteristics of the optimal full MAMS design, i.e. design scenario (*ii-1*) in Table 3 of the manuscript, under different configurations of treatment effect. The design (pairwise) power in all scenarios are 0.98 and 0.80 in stages 1 and 2, respectively. The design pairwise significance level for the selected arm at stage 2 analysis is $\alpha_{2S} = 0.009$ (one-sided) in all scenarios. Number of simulations is 250,000 in each experimental condition. SS, sample size; FWER, familywise type I error rate, calculated under the global null hypothesis.

❀) Any-pair power is the probability of showing a statistically significant effect under the targeted effects for at least one comparison.

✻) All-pairs power is the probability of showing a statistically significant effect under the targeted effects for all comparison pairs.

F2: Power of the 4-arm 2-stage RED MAMS selection design

Scenario	No of arms		Assumed effect sizes $(\theta_1, \theta_2, \theta_3)$	Max. SS	Pr. of selecting any effective arm	Power to identify any effective arm	FWER
	Selected	Effective					
1	1	1	$-0.05, 0, 0$	2282	0.88	0.78	0.025
2	1	2	$-0.05, -0.025, 0$	2282	0.96	0.76	0.025
3	1	2	$-0.05, -0.05, 0$	2282	0.98	0.90	0.025
4	1	3	$-0.05, -0.025, -0.025$	2282	1.00	0.74	0.025
5	1	3	$-0.05, -0.05, -0.025$	2282	1.00	0.87	0.025
6	1	3	$-0.05, -0.05, -0.05$	2282	1.00	0.93	0.025

Table 3. The operating characteristics of the chosen RED MAMS selection design, i.e. design scenario (*i-2*) in Table 3 of the manuscript, under different configurations of treatment effect. The design (pairwise) power in all scenarios are 0.95 and 0.88 in stages 1 and 2, respectively. The design pairwise significance level for the selected arm at stage 2 analysis is $\alpha_{2S} = 0.015$ (one-sided) in all scenarios. Number of simulations is 250,000 in each experimental condition. SS, sample size; FWER, familywise type I error rate, calculated under the global null hypothesis.

Appendix G: The operating characteristics of the 3-stage RED trial

This is the first presentation of MAMS selection design within the general MAMS framework introduced by Royston et al. In principle, the design can be extended to have further interim analyses.

In this section, we present the operating characteristics of the 3-stage RED trial with treatment selection in stage 1 and lack-of-benefit analysis at stage 2. We extended the the 2-stage RED trial design, i.e. design scenario (*i-2*) in Table 3 of the manuscript, by adding a further interim (lack-of-benefit) analysis after the selection stage. The results are presented in Table 4 below. The top section of the table presents the design specification in this 3-stage setting. The bottom section presents the familywise type I error rate (FWER) and overall power of the 3-stage design, calculated using simulations.

In this setting, the research arm with the largest treatment effect in stage 1 is selected to continue to the next stage. The selected research arm will be compared against the control arm in a lack-of-benefit analysis at stage 2. This occurs at 50% control arm information time, i.e. when the outcome data is observed for half of the total sample size for the selected comparison. The (one-sided) significance level and (unconditional) design pairwise power for this analysis are 0.235 and 0.95, respectively. If the observed p-value at stage 2 is larger than the pre-specified threshold of 0.235, the trial can be stopped for lack of sufficient activity. This analysis has (unconditional) pairwise power of 0.95 under the alternative hypothesis. If the selected arm passes the lack-of-benefit analysis at stage 2, it continues to the final stage. The final primary analysis is carried out when the primary outcome data is observed for 1946 (2x973) individuals in the two arms that have reached the final stage. The primary null hypothesis is rejected at the final stage if the observed p-value is smaller than the final stage significance level of 0.015.

The simulation results indicate that the loss in the overall power is minimal under this design, reducing to 0.77 from 0.78. The FWER is 0.0246 under the global null hypothesis when the lack-of-benefit stopping rule is considered as 'binding'. However, the strong control of the FWER requires its calculation under the non-binding stopping rules which is still 2.5% (one-sided).

Stage (j)	Adaptation/analysis	No of comparisons	Outcome	Sig. level (α_j) (one-sided)	Design power (ω_j)	Control arm sample size
1	Treatment selection	3	MMS	0.600	0.95	168
2	Lack-of-benefit analysis	1	MMS	0.235	0.95	488
3	Primary efficacy analysis	1	MMS	0.015	0.88	973
**Overall operating characteristics:**						
Familywise type I error rate (FWER)			0.025			
Overall power			0.77			
Probability of correct selection			0.88			
Maximum sample size			2282			

Table 4. The design specification and operating characteristics of 4-arm 3-stage RED (MAMS) selection design with treatment selection at stage 1 and lack-of-benefit analysis at stage 2. The target effect size is -0.05 in absolute terms from the control arm event rate of 0.15. Number of simulations is 250,000. MMS, maternal mortality or invasive surgical procedure.

References

1. Choodari-Oskooei B, Sydes M, Royston P, Parmar MK. Multi-arm multi-stage (MAMS) platform randomized clinical trials (book chapter), Principles and Practice of Clinical Trials. 1st ed. Springer; 2022. Available from: https://doi.org/10.1007/978-3-319-52677-5_110-1.
2. Blenkinsop A, Parmar MKB, Choodari-Oskooei B. Assessing the impact of efficacy stopping rules on the error rates under the MAMS framework. Clinical Trials. 2019;16(2):132–142.
3. Dunnett CW. A Multiple Comparison Procedure for Comparing Several Treatments with a Control. Journal of the American Statistical Association. 1955;50(272):1096–1121.
4. Jung SH, Lee T, Kim K, George SL. Admissible two-stage designs for phase II cancer clinical trials. Stat Med. 2004;23(4):561–9.
5. Wason J, Stallard N, Bowden J, Jennison C. A multi-stage drop-the-losers design for multi-arm clinical trials. Statistical Methods in Medical Research. 2017;26(1):508–524.
6. Jennison C, Turnbull BW. Confirmatory Seamless Phase II/III Clinical Trials with Hypotheses Selection at Interim: Opportunities and Limitations. Biometrical Journal. 2006;48(4):650–655. Available from: http://doi.wiley.com/10.1002/bimj.200610248.
7. Friede T, Stallard N. A comparison of methods for adaptive treatment selection. Biometrical Journal. 2008;50(5):767–781.
8. Choodari-Oskooei B, Bratton DJ, Gannon MR, Meade AM, Sydes MR, Parmar MK. Adding new experimental arms to randomised clinical trials: Impact on error rates. Clinical Trials. 2020;17(3):273–284. Available from: https://doi.org/10.1177/1740774520904346.
9. Stallard N, Kunz CU, Todd S, Parsons N, Friede T. Flexible selection of a single treatment incorporating short-term endpoint information in a phase II-III clinical trial. Statistics in Medicine. 2015;34(23):3104–3115.
10. Stallard N, Todd S. Sequential designs for phase III clinical trials incorporating treatment selection. Statistics in Medicine. 2003;22:689–703.
11. Lu X, He Y, Wu SS. Interval estimation in multi-stage drop-the-losers designs. Statistical Methods in Medical Research. 2018;27(1):221–233. Available from: https://journals.sagepub.com/doi/pdf/10.1177/0962280215626748.
12. Kunz CU, Friede T, Parsons N, Todd S, Stallard N. Data-driven treatment selection for seamless phase II/III trials incorporating early-outcome data. Pharmaceutical Statistics. 2014;13(4):238–246.
13. Kunz CU, Friede T, Parsons N, Todd S, Stallard N. A comparison of methods for treatment selection in seamless phase II/III clinical trials incorporating information on short-term endpoints. Journal of Biopharmaceutical Statistics. 2015;25(1):170–189.
14. Althabe F, Aleman A, Tomasso G, Gibbons L, Vitureira G, Belizan JM, et al. A pilot randomized controlled trial of controlled cord traction to reduce postpartum blood loss. International Journal of Gynecology Obstetrics. 2009;107(1):4–7. Available from: 10.1016/j.ijgo.2009.05.021.
